# Nurse-led active surveillance for prostate cancer is safe, effective and associated with high rates of patient satisfaction—results of an audit in the East of England

**DOI:** 10.3332/ecancer.2018.854

**Published:** 2018-07-25

**Authors:** Estelle Martin, Satyendra Persaud, John Corr, Rowan Casey, Rajiv Pillai

**Affiliations:** 1 Department of Urology, Colchester General Hospital, Essex CO4 5JL, UK; 2 Department of Urology, Broomfield Hospital, Essex CM1 7ET, UK

**Keywords:** nurse-led, active surveillance, prostate cancer

## Abstract

**Introduction:**

Active surveillance (AS) is an option in the management of men with low-stage, low-risk prostate cancer. These patients, who often require prolonged follow-up, can put a strain on outpatient resources. Nurses are ideally placed to develop advanced roles to help meet this increased demand—a model we have utilised since 2014. We set about to comprehensively evaluate our nurse-led AS (NLAS) programme.

**Patients and methods:**

An audit of patient notes was carried out to assess compliance with trust and national guidelines. A questionnaire was designed to capture patients’ experiences of NLAS. This was piloted and then distributed to all patients in our NLAS programme. A second questionnaire was designed to assess the views of stakeholders within the department.

**Results:**

Compliance with various aspects of local guidelines ranged from 88.8% to 100%. 143 patients are currently in the programme with a mean duration of AS of 37.03 months. 104 questionnaires were returned. Most of the patients were aware of the role of the nurse prior to their visit, and all were happy to meet with a nurse. All of the patients indicated their confidence in the nurse monitoring their prostate-specific antigen. Among those requiring further investigations, 85.3% were happy with the information they received prior to their tests. Overall, 96.2% were either very satisfied or satisfied with NLAS. All stakeholders held positive views about NLAS.

**Conclusions:**

NLAS is safe and effective. Patients and stakeholders alike held positive views of the programme.

## Introduction

Prostate cancer (PCa) is the most common cancer among males in the UK with 47,000 new cases diagnosed annually [1]. Most men with early PCa are asymptomatic, and it is widely known that the incidence of PCa outstrips its mortality, meaning that the chance of being diagnosed with PCa is approximately six times the chance of dying from it [2].

Although the benefit of screening is controversial, many asymptomatic men are still screened, diagnosed and treated for low-grade low-risk PCa which may never have become clinically evident and in the process are exposed to a number of potential complications [3]. Active surveillance (AS) aims to avoid unnecessary over-treatment of clinically insignificant PCa [4, 5]. It involves the postponement of invasive treatment accompanied by careful disease surveillance. If disease progression is evident, or a patient has a change in treatment preference, treatment can still be offered with curative intent [6]. AS is becoming an increasingly popular initial management strategy among urologists [3]. In the UK, offering AS to patients with low-volume, low-risk PCa is deemed a best practice [7]. This management strategy benefits the patients as it postpones radical treatment and the associated side effects [8]. Other benefits include quality-of-life gains for the patient with potentially lower overall healthcare costs [9, 10]. In AS, PCa is monitoring through prostate-specific antigen (PSA), digital rectal examination (DRE) and biopsies/imaging at specific intervals with management strategies in place should disease progression become evident [11].

Because patients on AS have indolent tumours and are likely to require protracted follow-up, a strain on outpatient resources results by filling clinics with patients who more often than not require basic follow-up rather than expert treatment planning [12, 13]. It was recognised in 2006 that nurses were ideally placed to develop advanced roles to help meet this increased demand [14]. This has been particularly useful as health services try to maintain standards of patient care and satisfaction in an era of austerity, working time directives and waiting time targets. This evolving role for nurses has been advocated by many government papers including Department of Health [15] Cancer Reform Strategy and Department of Health [16] high-quality care for all.

Despite the widespread support for nurse-led AS (NLAS) clinics among British urologists [17], most of the patients are still being managed in general urology clinics [18]. Our Trust has been supportive of nurse-led clinics, and our NLAS clinic was commenced in 2014. It is currently run by a single-trained advanced nurse practitioner (ANP) and cases are referred by a senior clinician or via our multidisciplinary team (MDT) based on a set local protocol (Appendix). Patients breaching surveillance protocols are flagged to the consultant. Our programme, we feel, is a success and can be reproduced at other centres in the United Kingdom and beyond. This paper represents our first attempt to audit and evaluate this service from both patient and practitioner points of view.

## Materials and methods

First, an audit of patients’ notes was undertaken on all patients enrolled in the NLAS programme as outlined below. Notes were assessed to determine compliance of the NLAS clinic with local AS guidelines.

A questionnaire was then designed to consider patients’ experiences of NLAS. The questionnaire represented the patient’s journey through the programme and included five main sections—the patient’s experience around diagnosis, whether NLAS was explained sufficiently, their experience of NLAS itself, the tests and investigations involved and the demographics of the population. The questionnaire was adapted from the National Health Service (NHS) National Cancer Patient Experience Survey [19], the Prostate Care Questionnaire for Patients and Carers [20] as well as the Trusts own service evaluation questionnaires. There was also space provided for the patients to expand on their answers or give them context if required. The questionnaire was piloted with patients at a PCa support group meeting, adjusted and was then sent to a random sample of ten NLAS patients for a second round of piloting. Questionnaires were then mailed to patients in the study and a stamped addressed envelope so that completed questionnaires could be mailed in anonymously.

Patients were included in the audit and questionnaire evaluation if they had been referred to and were still receiving care through NLAS since its inception in June 2014 to June 2017. It was calculated that the number needed to participate for the results to reach a confidence level of 95% with a confidence interval of ±5% (Creative Research Systems 2012) was 104. This number was attained, reaching the figure required for statistical significance. However, given that answers were missing for several questions, power of the data was likely to have been reduced, and for this reason, the data were analysed using descriptive statistics.

A second questionnaire was designed to evaluate stakeholders’ (referring clinicians) views on NLAS. This questionnaire was adapted from the Protec-T studies evaluation of NLAS [21]. The questionnaire was piloted with one Stakeholder and minor amendments made to the wording to increase clarity. This questionnaire was distributed to all six consultants and five registrars involved in referring to NLAS.

## Results

Of 143 patients in the program, four were excluded from the notes audit due to missing data. Overall patients’ age at commencement of AS ranged from 46 to 76 years old; mean 65.9 ± 5.98 years. AS duration ranged from 7 to 156 months (mean 37.03 months) and NLAS duration ranged between 1 and 36 months with a mean of 14.27 ± 8.93 months. In total, 119 (85.6%) patients met national eligibility criteria and 132 (95%) local. The 143 patients receiving NLAS were seen a total of 417 times.

Table 1 shows compliance with essential aspects of local AS guidelines. Of the 417 clinic appointments, 100% occurred at the correct interval, and 100% addressed patients’ PSA. In 97.6% of patients, lower urinary tract symptoms (LUTS) were addressed. The least compliance was with digital rectal exams where only 88% compliance was achieved.

### Patient evaluation questionnaire

All 143 patients were sampled of which 104 responses were returned, a response rate of 72.7% (95% CI). Patient ages ranged from 52 to 82. The majority of patients had been on AS for 2 (23.1%) or 3 years (23.1%) and the majority had met with the ANP four times or more (Table 2).

Before their initial NLAS appointment, 86 (82.7%) patients were aware of the ANP role. 104 (100%) patients were happy seeing the ANP as opposed to the doctor, stating she introduced herself and explained her role well. 104 (100%) were comfortable talking to the ANP, with 101 (97.1%) feeling able to discuss all aspects of their condition. Of those who responded, 102 (100%) felt the ANP met their needs for follow-up. 103 (100%) had confidence and trust in the ANPs skills and ability and felt she treated them with dignity and respect. Overall, of those who completed the questionnaire, 100% had confidence in the ANP monitoring their PCa.

When asked about regular testing/monitoring for PCa, 97.1% (99/102) understood the rationale for this, with 100% of those who answered feeling the ANP explained the results well. In the last year, 34 (32.7%) stated they needed further investigations including magnetic resonance imaging (MRI) and/or biopsy. Among this subgroup, 85.3% (29) were satisfied with the information that they received prior to the procedure but 8.8% (3) would have liked more. However, all patients who completed the question felt the results were explained fully.

Of those who answered, 61 (62.9%) preferred seeing the ANP instead of the consultant, 4 (4.1%) preferred to see the consultant and 32 (33%) were not sure (seven did not answer). Overall 101 (96.2%) patients were either very satisfied or satisfied with NLAS, two (1.9%) neither satisfied nor dissatisfied and one (1%) did not answer.

The patients felt they had more time with the ANP in NLAS rather than seeing a consultant, with 98.1% feeling enough time was given to address their concerns/issues and nine patients commenting further on this. 100% of patients were comfortable taking to the ANP.

With regard to receiving their diagnosis, 100 (96.2%) patients understood their diagnosis, two (1.9%) patients did not understand and two (1.9%) could not remember. When making treatment decisions, 98 (94.2%) patients understood their treatment options, four (3.8%) did not have their treatment options explained to them and two (1.9%) could not remember. 91 (87.5%) patients felt that the risks of AS were explained to them effectively, with 91 (87.5%) patients receiving written information regarding AS. 11 (10.6%) claimed not to have received written information.

116 freehand comments were made. The table below details the topics related to NLAS covered by the remaining comments (Table 3). It should be noted that there were no dropouts due to concerns over the clinic being nurse led. Also, there were no serial nonattenders. Overall compliance with the clinics was excellent.

### Stakeholder questionnaire

The questionnaires were distributed to 11 clinicians of whom ten responded. The length of the time stakeholders had been involved in AS ranged from 1 to 20 years with a mean of 12 years 1 month. The majority of stakeholders were aware that there was a departmentally agreed AS protocol; however, 20% were not. All respondents were aware of Trust’s NLAS service and 100% agreed it provided high-quality psychological support and clinical care. All stakeholders strongly agreed NLAS provided continuity of care whilst reducing the burden in standard NHS clinics. The majority of respondents (70%) felt NLAS was cost-effective.

## Discussion

This service evaluation found high levels of patient and stakeholder satisfaction and good alignment with national and local standards for AS. In general, the audit demonstrated good compliance with national and local standards. When considering national eligibility criteria, 85.6% of referrals were compliant. This mirrors the findings of Phillipou et al [18] who reviewed AS nationally and found NICE (2014) guidelines were generally being followed. Considering the local protocol, 95% of patients met eligibility criteria with seven patients falling outside the eligibility criteria as their PSA was over 15 ng/mL. This should be taken in context that the PSA threshold as recommended by NICE (20 ng/mL) is higher than our local protocol. In all other elements of local eligibility guidelines, disease stage and Gleason grade, the service was 100% compliant. If the local eligibility criteria for PSA were amended in keeping with national guidelines, only one patient would have fallen outside eligibility criteria meaning compliance would have been 99.3%. Although this patient’s PSA was over 20 ng/mL at diagnosis he went on to have a transurethral resection of the prostate (TURP), and at the point of referral to NLAS, his PSA was actually 9 ng/mL.

MRI is recommended at enrolment to AS by NICE, but no recommendation is made in local guidelines. Nonetheless, the local service was compliant with MRI at enrolment in 92.6% of cases. Of the ten patients who did not receive an MRI, three have since had one done and five were diagnosed incidentally following TURP as opposed to being referred for an elevated PSA in which case they would have had the MRI prior to biopsy.

Although NLAS was 100% compliant with clinic intervals and PSA evaluation, compliance with DRE at each visit was only 88.8%. In the initial phase of the NLAS clinic, it was registrar-led and DRE was often excluded without a clinical reason documented and based on clinical judgement. Since December 2015, when the ANP assumed the lead, all patients have had the DRE undertaken unless the patient declined or there was a clinical rationale, as per the local protocol.

Service users are key players in determining whether their experience of care is of acceptable quality and the patient’s perspective is therefore crucial [22]. Wade identified three key themes in support of care which is nurse-led including quality of care, efficient use of resources and convenience of care [21]. In our study, four themes specifically relating to NLAS emerged from the freehand comments including the timing and time allowed for NLAS, continuity of care, freeing up consultants to deal with more complex cases and the patient’s confidence in NLAS.

Consistency was identified as a key issue—patients seem to place value on the rapport developed with the same clinician. Jones [23] noted that 93% of patients cited this as important. Lewis et al [24] and Faithful et al [25] noted that continuity led to men who received nurse-led care being significantly more satisfied, as it enables them to build trusting long-term relationships with healthcare providers. For example, in our NLAS programme one ANP facilitates the clinic and patients value this continuity, with seven specifically commenting on this.

Overall patients had very positive views about NLAS. Similarly, in an analysis of the Protec-T trial, where NLAS was widely utilised, the authors noted that men held very positive views of NLAS—they expressed confidence in the quality of care and perceived the programme to be cost-effective and convenient [21].

The idea that NLAS has led to more efficient use of outpatient time has previously been noted by several authors. Lane and Minns [26] as well as Faithful et al [25] found that nurse-led care provides more efficient outpatient care and decreases waiting times. This was also a significant finding among patients in the Protec-T trial where patients believed that nurse-led care was an efficient use of resources [21]. Nurse-led clinics can decrease patient numbers in consultant clinics and allow more time for individual consultations, decreasing waiting time [27].

Freeing up consultant time and reducing pressure on outpatient services was another frequently occurring point. Clinicians in this study also highlighted this, with all nurses and urologists questioned agreeing NLAS benefited the patient, providing continuity, flexibility and high-quality care and reducing pressures within the NHS. Frew and Leung [28] state their move to a nurse-led model provided in secondary care freed consultant time, although it should be noted that this was a service originally provided in the community with practice nurses administering hormone injections to patients in the general practitioner's practice or their home. McGlynn et al [29] also state their nurse-led model reduced outpatient pressure for appointments and freed up consultant capacity.

NLAS patients reported high levels of confidence in the nurse-led service with 22 specifically commenting on this. This has previously been described by several authors with nurse-led clinics both in PCa diagnosis and treatment [12, 21, 30, 31]. This adds to the growing body of evidence suggesting nurse-led follow-up is high-quality and safe, and patients benefit from the continuity and easy access to support. Our study has reaffirmed that this also holds true in the setting of AS. In this area, there is a paucity of data.

The patient evaluation questionnaire did identify some areas for improvement. A minority of patients did not feel that that information was relayed in a satisfactory manner. Only 86 (82.7%) patients were aware of the ANP role prior to being seen in NLAS. The latter issue in particular may potentially be reduced by providing patients with literature on NLAS at the time of treatment decision and ensuring that they understand all that follow-up entails.

With regard to stakeholders, 100% of clinicians felt NLAS provided continuity. This view was shared by stakeholders in the ProTec-T trial [21]. They also felt that NLAS relieved the pressure on outpatient clinics. It has been estimated that nurse-led clinics may result in as much as 30% reduction in outpatient workload [32]. This also means that doctors may focus on more complex patients requiring specialist intervention. The stakeholders also felt that NLAS was cost-effective. While an economic analysis did not form part of our assessment, Faithfull et al [25] noted that nurse-led follow-up has the potential to cut follow-up costs by as much as 31%. The most commonly cited skill stakeholders felt would be beneficial for the ANP to undertake was performing prostate biopsies to further improve continuity; however, training was identified as the main barrier to this. The ANP is aware of a number of nurses nationally who undertake prostate biopsies and is working with them to see how competency in this skill can be gained.

This evaluation did have potential limitations. First, we only considered patients currently enrolled in our programme and did not take into account those men who would have left the programme nor the reason for which they left. Second, patients may have been reluctant to criticise a programme in which they are currently enrolled. Last, we have only evaluated our Trust and so probably cannot be generalised to other sites.

## Conclusion

With increasing diagnosis of PCa globally, and in particular, in times of austerity, strategies geared towards efficient use of personnel are of paramount importance. Nurse-led care is becoming an increasingly common model. This service evaluation supports the value of nurse-led care both to patients and the health services. Our take-home message is that nurse-led care is here to stay and is both safe and effective. We believe that this model could easily be reproduced at other centres in the United Kingdom or beyond.

## Conflicts of interest

The authors declare that they have no competing conflicts of interest

## Funding declaration

The authors declare that they received no external funding of any sort for this research project

## Figures and Tables

**Table 1.** Compliance with essential aspects of local AS guidelines.

Clinic outcome	Standard	Target	No. of applicable appointments	No. (%) appointments compliant
**Interval between clinic appointments appropriate**	Patients are seen 3–6 monthly for the first 2 years then 6 monthly	**100%**	**417**	417(100%)
**LUTS addressed**	Patients LUTS should be addressed at every appointment	**100%**	**417**	407(97.6%)
**PSA discussed**	PSA should be reviewed at every appointment	**100%**	**417**	417(100%)
**DRE performed**	DRE should be performed at a minimum of 6 monthly intervals	**100%**	**374—**15 appointment episodes excluded as DRE not performed for clinical reasons; 28 excluded as patient refused	332(88.8%)
**Total no. of appointments where criteria met**		**100%**	**374**	**327(87.4%)**

**Table 2.** Number of times reviewed in NLAS.

Number of occasions patient been seen in NLAS	Number of patients (%)
Once	10(9.6%)
Twice	19(18.2%)
Three times	27(26%)
Four or more	47(45.2%)
Not answered	1(1%)

**Table 3.** Freehand comments among NLAS patients surveyed.

Main theme of the comment	Number of comments
Compliments/thanks	39
Confidence in ANP	22
Length of appointment/feeling unhurried	9
Continuity	7
ANP easier to talk to	5
Appointments ran to time	4
Frees up consultants to see more complex patients	4
When will I next see consultant	3

## Appendix.

**Table:** Summary of local AS protocol.

No.	Nurse Assessment	Rationale	Indications for referral to consultant urologist/oncologist
3.1	General enquiry regarding current health status (and sexual function/ability, where appropriate)	To identify deterioration in patients general well-being and development of symptoms suggestive of metastatic disease	Altered mental/physical function or status pertinent to prostate condition Bone pain Lower limb swelling/weakness Altered limb sensation
3.2	Assess LUTS by either review of International Prostate Symptom Score by direct patient questioning	To identify if LUTS are significant and compromising quality of life requiring potential change of treatment plan, i.e., initiation of medication or surgery	LUTS not responding to medication or requiring initiation or alteration of treatment
3.3	If LUTS deteriorated perform flow rate, post residual volume, frequency volume chart and urinalysis	To identify nature of LUTS To identify irritative symptoms To identify urinary tract infection/painless haematuria	Obstructive flow pattern PVR > 300 mL Irritative symptoms—for Consultant review Signs and symptoms of infection to discuss need for antibiotics Positive result for haematuria
3.4	Review of PSA result and any other biochemical results ordered and available at consultation	To identify PSAdt, disease progression and potential need for change in treatment plan/referral back to MDT for re-discussion or re-biopsy	PSAdt < 12 months Three consecutive increases in PSA levels Abnormal U&Es, liver function tests and bone profile tests
3.5	DRE at 6/12 intervals or if LUTS deteriorated	To identify alteration in size/texture of gland	New abnormality detected/nodule felt
3.6	Discuss all findings with patient ± spouse/partner/carer and offer copy of clinic letter Document all findings	To ensure patient fully understands disease status and intended plan of care To ensure compliance with Nursing and Midwifery Council record keeping (2008)	Patient preference for change in treatment plan
3.7	Arrange 2 yearly transrectal ultrasound/template biopsies or sooner if PSA/DRE deteriorates ± MRI	To ensure no change to disease volume of grade that requires intervention	If change to disease grade or volume or for patient anxiety reasons
3.8	Arrange follow-up appointment and provide form for PSA test prior to next appointment	To ensure disease monitored in accordance with British Association of Urological Nurses guidelines (2008) i.e.: 3–6 monthly for minimum of 2 years for active monitoring patients, 6 monthly for Watchful Waiters then 6 monthly after 2 years of stable active monitoring	Change in clinical condition of patient Patient preference To agree 6 monthly review schedule
